# Effects of venoms on neutrophil respiratory burst: a major inflammatory function

**DOI:** 10.1590/1678-9199-JVATITD-2020-0179

**Published:** 2021-06-28

**Authors:** Jamel El-Benna, Margarita Hurtado-Nedelec, Marie-Anne Gougerot-Pocidalo, Pham My-Chan Dang

**Affiliations:** 1 Université de Paris, INSERM-U1149, CNRS-ERL8252, Centre de Recherche sur l’Inflammation (CRI), Laboratoire d’Excellence Inflamex, Faculté de Médecine Xavier Bichat, Paris, France.; 2 AP-HP, Centre Hospitalier Universitaire Xavier Bichat, UF Dysfonctionnements Immunitaires, Paris, France.

**Keywords:** Neutrophils, Venom, Inflammation, ROS, NADPH oxidase, PLA2, L-amino acid oxidase, Mastoporan, Parabutoporin, Disintegrins

## Abstract

Neutrophils play a pivotal role in innate immunity and in the inflammatory response. Neutrophils are very motile cells that are rapidly recruited to the inflammatory site as the body first line of defense. Their bactericidal activity is due to the release into the phagocytic vacuole, called phagosome, of several toxic molecules directed against microbes. Neutrophil stimulation induces release of this arsenal into the phagosome and induces the assembly at the membrane of subunits of the NAPDH oxidase, the enzyme responsible for the production of superoxide anion that gives rise to other reactive oxygen species (ROS), a process called respiratory burst. Altogether, they are responsible for the bactericidal activity of the neutrophils. Excessive activation of neutrophils can lead to extensive release of these toxic agents, inducing tissue injury and the inflammatory reaction. Envenomation, caused by different animal species (bees, wasps, scorpions, snakes etc.), is well known to induce a local and acute inflammatory reaction, characterized by recruitment and activation of leukocytes and the release of several inflammatory mediators, including prostaglandins and cytokines. Venoms contain several molecules such as enzymes (phospholipase A2, L-amino acid oxidase and proteases, among others) and peptides (disintegrins, mastoporan, parabutoporin etc.). These molecules are able to stimulate or inhibit ROS production by neutrophils. The present review article gives a general overview of the main neutrophil functions focusing on ROS production and summarizes how venoms and venom molecules can affect this function.

## Background

Polymorphonuclear neutrophils (PMN) are the most abundant circulating leukocytes as they normally constitute 60 to 70% of white blood cells [1]. PMN have a key role in host defense against microbes as they are the first cells to migrate out of the circulation by a process called chemotaxis and are massively recruited at the infection site [2-5]. Once at the infection site, neutrophils recognize the pathogen *via* different receptors expressed at their cell surface, followed by engulfment of the microbe into a vacuole called the phagosome or phagolysosome [6-9]. Microbes are then killed by PMN through the release into the phagosome of highly toxic agents such as reactive oxygen species (ROS) and granule contents such as myeloperoxidase (MPO), glucosidases, proteases and anti-bacterial peptides [10,11]. Once the microbe is killed, neutrophils die by apoptosis, after which they are phagocytized and eliminated by the local macrophages through a process called efferocytosis, thereby cleaning the infection site. Thus, PMN are anti-inflammatory components of the innate immune system as their physiological role is to resolve the infection and the inflammation. Nevertheless, when PMN are excessively activated, the “cleaning task” cannot be completed and they become harmful to the surrounding tissues as they can induce cell injury and modification of cell homeostasis, metabolism and signaling [12-14].

Envenomation is a process by which a venom is inoculated into an organism by the bite or sting of different animal species (bees, wasps, scorpions, snakes etc.), inducing a localized inflammatory reaction characterized by the usual symptoms or redness, pain, heat and swelling, and in some cases, triggering an allergic response that can lead to death [15-17]. Venoms consist of a mixture of toxic agents with different properties and actions [18-20]. A large number of toxic venom agents has been characterized, and consists of peptides and proteins that can modify host cells. They include phospholipase A2 (PLA2) that cleaves plasma membrane phospholipids to release arachidonic acid; L-amino acid oxidase (LAAO) that catalyzes the deamination of L-amino acids to the corresponding a-ketoacids and production of hydrogen peroxide and ammonia; metalloproteinases that degrade membrane proteins; a family of peptides called disintegrins that bind to various cellular integrins; mastoparan that stimulates heterotrimeric G-proteins (Gi) and upregulate cellular functions; and parabutoporin thyat has antimicrobial properties and can modulate cell functions. These molecules are known to induce a variety of immune responses, including mastocyte degranulation, T cell activation, inflammasome activation in macrophages, and neutrophil activation [21-24]. In this review, after an overview of neutrophil ROS production, a key inflammatory function, we will summarize the most characterized effects of venom components on this neutrophil function and the known mechanism of action.

### Recruitment of neutrophils to the infection site and their activation

**Figure 1.**
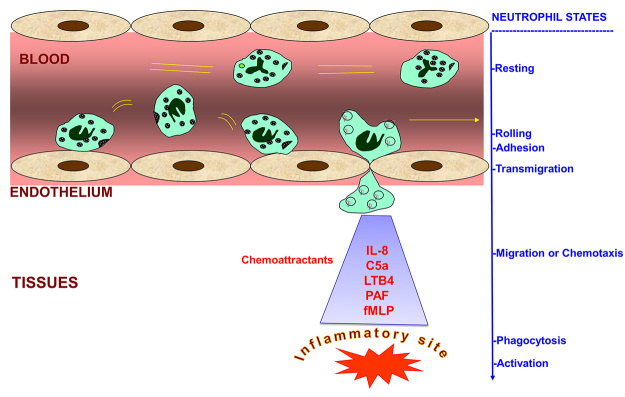
Migration of neutrophils from blood to the inflammatory site. Circulating neutrophils are in a resting state, also known as the dormant state. Upon inflammation, neutrophils start rolling, adhere and migrate to the inflammatory site, attracted by a multitude of chemoattractants such as IL-8, C5a, LTB4, PAF and fMLP.

Upon infection, keratinocytes, epithelial cells, tissue resident macrophages, and dendritic cells produce several soluble agents such as lipid mediators (platelet-activating factor (PAF), leukotriene B4 (LTB4), etc.), and several cytokines (IL-1, IL-8, IL-17, TNFα, etc.), which along with agents released by the pathogen (LPS, toxins, etc.), induce endothelial cell stimulation [6-8]. These agents promote the expression of E- and P-Selectins on the surface of endothelial cells. Resting circulating PMN detect these selectins via their respective ligands (L-selectin; CD62L) and start rolling onto the endothelial cells. Stimulated endothelial cells then express intercellular adhesion molecule-1 (ICAM-1), molecules that are recognized by neutrophil integrins (CD11b/CD18) and induce firm adhesion of the neutrophils to the endothelial cells. PMN then transmigrate through the endothelial cell junctions and move into the tissues towards the infection site, attracted by several chemoattractants such as PAF, LTB4, IL-8, the C5a fraction of the complement and the bacterial peptide fMLP (N-formyl-methionyl-leucyl-phenylalanine) (Figure 1). These chemoattractants induce signaling pathways that result in polarization of PMN and actin polymerization at the cell leading edge, positioning them towards the gradient of chemoattractants [6]. Chemotaxis is mainly controlled by the PI3Kinase and p38MAPKinase pathways, and by small G proteins such as Rac1 and Rac2 [6].

Once at the infectious site, PMN recognize microbe motifs via receptors of the Toll family [Toll-like receptors (TLR)] [25, 26]. Human neutrophils express several TLR receptors that recognize various ligands, including TLR1 (recognizes lipoproteins), TLR2 (recognizes peptidoglycans from bacteria and fungi), TLR4 (recognizes LPS), TLR5 (recognizes flagellin), TLR6 (recognizes mycoplasma lipoprotein), TLR7 and TLR8 (recognize single strand virus RNA), and TLR9 (recognizes CpG bacterial DNA) [25, 26]. These TLR agonists along with pro-inflammatory cytokines and agents found at the inflammatory site induce pre-activation of the neutrophils, a process called priming, which accelerates the phagocytosis of the microbe and its killing [12, 27, 28]. The binding of PMN to the microbe occurs through various opsonins such as the immunoglobulins G (IgG), which bind to FcgRIIA/CD32 and FcgRIIIB/CD16b, and the C3b and C3bi proteins produced by activation of the complement, which bind to CR1/CD35 and CR3/CD18+CD11b, respectively [3, 11]. The recognition is generally followed by engulfment of the microbe, which becomes surrounded by the membrane envelope, ultimately forming a vacuole called the phagosome or phagolysosome. Engulfment of the microbe triggers the PMN killing process that engages proteases, ROS and other toxic agents, leading to the death and destruction of the pathogen [3, 10, 11]. 

### Neutrophil arsenal of toxic agents

In resting cells, PMN toxic agents are stored in different granules that have different composition and density [29, 30]. The most dense granules are called azurophil or primary granules as determined by Percoll-gradient ultracentrifugation, the specific granules or secondary granules are less dense than the former. Followed by the tertiary granules, also called gelatinase granules for their large content in gelatinase, and finally, the highly mobilizable secretory vesicles contain mainly plasma proteins. The detailed content of these granules is described in Table 1. The release of these granule contents upon cell activation is called degranulation and is an important neutrophil function for host defense against pathogens and inflammation [9, 11]. Degranulation is induced upon phagocytosis but also by soluble agonists such as fMLP, phorbol myristate acetate (PMA), or calcium ionophores. Degranulation also allows expression of different receptors and the NADPH oxidase NOX2 at the cell membrane. It is controlled mainly by intracellular calcium, protein kinases such as PI3Kinase, p38MAPKinase and PKC and small G proteins such as Rac1 [31, 32].

**Table 1.** Different human neutrophil granules and their contents [29, 30].

Azurophil granules or primary granules (very dense: +++)*	Specific granules or secondary granules (less dense: ++)*	Gelatinase granules or tertiary granules (light: +)*	Secretory vesicles (very light: -/+)*
**Matrix** Myeloperoxidase (MPO)** Lysozyme Elastase Cathepsins Proteinase-3 glucuronidase defensins BPI Azurocidin/CAP37 α-mannosidase β-glucuronidase β-glycerophosphatase N-acetyl-β-gucosaminidase **Membrane** CD63 CD68 V-type H+-ATPase	**Matrix** Lactoferrin** Lipocalin/NGAL** Lysozyme Collagenase Gelatinase Histaminase hCAP-18 Heparanase Sialidase VitaminB12-Binding protein β2-microglobulin **Membrane** CD11b/CD18 CD177 CD15 CD66 CD67 Gp91phox/p22phox FPR (fMLP-R) TNF-R Fibronectin-R Vitronectin-R VAMP-2 Laminin-R Urokinase-type plasminogen activator-R	**Matrix** Gelatinase** Acetyltransferase Lysozyme β2-microglobulin Acetyltransferase **Membrane** CD11b/CD18 CD177 Gp91phox/p22phox FPR (fMLP-R) Fibronectin VAMP2 V-type H+-ATPase Urokinase-type plasminogen activator-R	**Matrix** Plasma proteins** **Membrane** CD11b/CD18 CD14 CD16 CD45 Gp91phox/p22phox FPR (fMLP-R) SCAMP Alkaline phosphatase CR1 V-type H+-ATPase VAMP2 C1q-R Urokinase-type plasminogen activator-R DAF

*Density as obtained by Percoll gradient technique [29,30]

**The specific granule marker(s)

### ROS production by neutrophils

**Figure 2.**
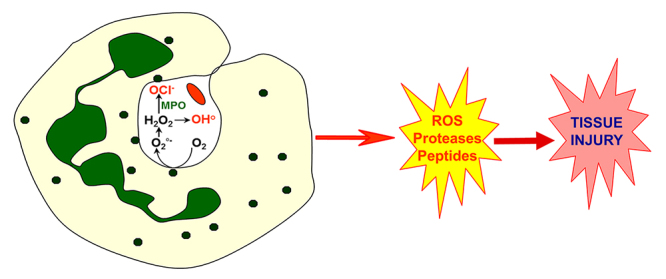
Activation of neutrophils. At the inflammatory site, neutrophils engulf the invading agent. Phagocytosis, in turn, induces a physiologically controlled activation of neutrophils, leading to the release of ROS and proteins inside the phagosome. However, excessive activation of neutrophils results in excessive release of ROS and granule contents in the extracellular space, contributing to tissue damage and inflammation.

Phagocytosis of a microbe stimulates PMN to produce ROS inside the phagosome (Figure 2). ROS include superoxide anion (O_2_^-^.), hydrogen peroxide (H_2_O_2_), hydroxyl radical (OH) and hypochlorous acid (HOCl) [10, 12, 33]. They are produced by phagocytes in a powerful process called "oxidative burst or respiratory burst", characterized by a rapid increase in oxygen and glucose consumption, and an abrupt ROS production. The first ROS molecule produced by PMN is superoxide anion (O_2_^-^), which is produced by the phagocyte NADPH oxidase through monovalent reduction of oxygen in the presence of an electron donor:

(**2 O_2_ + NADPH → 2 O_2_^-^ + NAD^+^ + H^+^**)

While superoxide is not the most reactive, it is essential for the production of other ROS and bacterial killing. O_2_^-^ is then transformed into H_2_O_2_ by dismutation in the presence of protons H^+^ (at acidic pH in the phagosome):

(**2O_2_2^-^ + 2H^+^ → H_2_O_2_ + O_2_**)

a reaction that can be catalyzed by superoxide dismutase (SOD) in other locations.

H_2_O_2_ and O_2_^-^ can react together through the Haber-Weiss reaction in the presence of a transition metal (or the Fenton reaction in the presence of iron) to generate hydroxyl radical (OH):

(**O_2_^-^ + H_2_O_2_ → or Fe^++^ or Cu^++^)( OH^°^ + OH^-^ + O_2_**),

Myeloperoxidase (MPO), released from azurophilic granules, catalyzes the transformation of H2O2 in the presence of a halogen (Cl^-^, Br^-^, I^-^) into very toxic molecules: 

(**H_2_O_2_^+^ H^+^Cl^-^ ( HOCl + HO)**.

The hypochlorous acid (HOCl) produced by this reaction reacts with amines resulting in chloramines:

(**H^+^ + OCl^-^ + R-NH_2_ → R-NHCl + HO**).

### Structure and activation of the phagocyte NADPH oxidase

**Figure 3.**
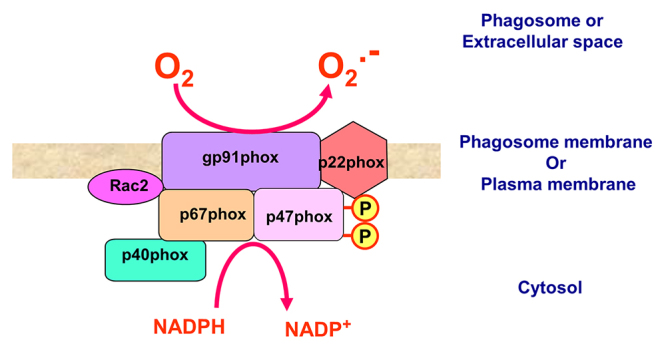
The NADPH oxidase complex. The active NADPH oxidase (NOX2) is composed of several cytosolic proteins (p67phox, p47phox, p40phox, rac2) and membrane-bound proteins (gp91phox and p22phox), initially referred to as cytochrome b558. The activated NADPH oxidase transfers an electron from the cytosolic NADPH to oxygen to form the radical, superoxide anion.

The enzyme responsible for the first step leading to ROS production is called the respiratory burst oxidase or the phagocyte NADPH oxidase (NOX2) [12, 33] which consists of several components, including the membrane cytochrome b558, a heterodimer composed of gp91phox/NOX2 and p22phox (phox: phagocyte oxidase), and the cytosolic p47phox, p67phox, p40phox and either Rac1 (in monocytes) or Rac2 (in neutrophils) (Figure 3). While dormant and spatially restricted in resting cells, the enzyme assembles at the membrane and becomes active to produce O2-. when the cells are stimulated. In intact cells, NADPH oxidase activation is accompanied by phosphorylation of almost all of its components (p47phox, p67phox, p40phox, gp91phox and p22phox) [34], which facilitates new protein-protein interactions and the assembly of the complex at the membrane of the phagosome. The vital importance of this enzyme is illustrated by a human genetic disorder called chronic granulomatous disease (CGD), which is due to gene mutation of one of the oxidase components (most frequently gp91phox and p47phox), and is associated with life-threatening bacterial and fungal infections [33]. However, excessive ROS release can also damage bystander host tissues (Figure 2), thereby amplifying inflammatory reactions [12-14].

**Figure 4.**
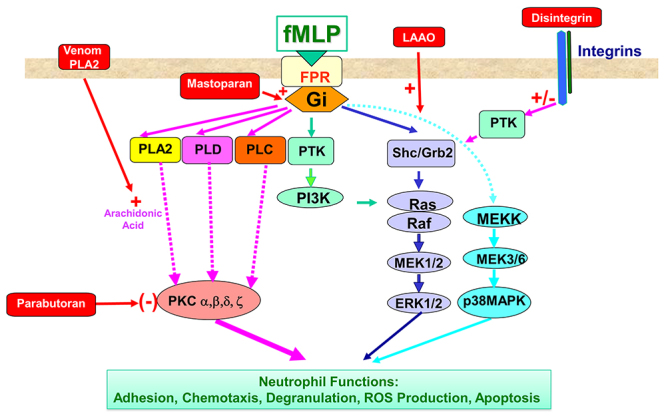
Molecular events underlying neutrophil activation and the effects of venom components. The fMLP peptide binds to its receptor called FPR (formyl peptide receptor), which activates heterotrimeric Gi proteins (proteins binding guanosine triphosphate, GTP) and tyrosine kinases. The G proteins then activate enzymes such as phospholipase C (PLC), phospholipase A2 (PLA2), phospholipase D (PLD), leading to the release of intracellular messengers, i.e., PLC catalyzes the formation of diacylglycerol (DAG) and inositol-triphosphate (IP3) from phosphatidylinositol 4,5-biphosphate (PIP2). IP3 is involved in the release of calcium from intracellular pools, while DAG activates protein kinase C (PKC). Activation of PLD results in phosphatidic acid production from phosphatidylcholine. Activation of PLA2 leads to the cleavage of membrane phospholipids to produce arachidonic acid, which can then be used as a substrate for leukotrienes and prostaglandins synthesis. FMLP induces activation of protein tyrosine kinases (PTK), which are upstream of the MAPKinase pathways (ERK1/2 and p38MAPKinase). All these kinases control neutrophil functions such as chemotaxis, degranulation, NADPH oxidase activation and apoptosis. The effect of venom components (PLA2, LAAO, disintegrin, mastoparan and parabutoparan) is shown in red.

NADPH oxidase activation in phagocytes can be induced by a large number of soluble and particulate factors such as opsonized bacteria, opsonized zymosan, formylated peptides such as (FMLP, C5a, PAF, calcium ionophores (ionomycin, A23187), and PKC activators like PMA [12]. The most studied agonists are FMLP and PMA. FMLP binds to its receptor, called FPR (formyl peptide receptor), which is a G-protein coupled receptor (GPCR) with seven trans-membrane domains [6, 35, 36]. The receptor activates heterotrimeric G proteins (proteins binding guanosine triphosphate, GTP) and protein tyrosine kinases (PTK). The G proteins then activate membrane enzymes such as phospholipase C (PLC), PLA2, and phospholipase D (PLD), leading to the release of intracellular messengers [6, 35, 36]. PLC cleaves a membrane lipid, phosphatidylinositol 4,5-biphosphate (PIP2) into diacylglycerol (DAG) and inositol trisphosphate (IP3). IP3 is involved in the release of calcium from intracellular pools, while DAG activates protein kinase C (PKC) [6, 35]. Activation of PLD results in phosphatidic acid production from phosphatidylcholine. Activation of PLA2 leads to the cleavage of phospholipids to produce arachidonic acid, which can then be used as a substrate for leukotrienes and prostaglandins synthesis. Neutrophil activation is accompanied by the activation of many protein kinases such as PTK, PKA, PKC, AKT and MAPKinase, which in turn phosphorylate many proteins with important cellular functions, including the NADPH oxidase components (Figure 4). In human neutrophils, various protein kinases have been implicated in the regulation of the NADPH oxidase activity, among them, the PKC family appears to play a major role after FMLF or PMA activation [34]. LPS and pro-inflammatory cytokines such as GM-CSF and TNFα, which alone do not activate NADPH oxidase but prime its activation by a secondary stimulus such as FMLP and C5a, induce partial phosphorylation of p47phox within a specific peptide sequence and upregulate NADPH oxidase assembly [12, 27, 28, 37]. Upon subsequent stimulation with FMLP or others, the phosphorylation of p47phox on multiple serines induces conformational changes and interaction of the SH3 domains of p47phox with the proline-rich region of p22phox, resulting in assembly of the active enzyme [12, 38].

### Effects of crude venoms on neutrophil ROS production

Venoms from different sources (bees, wasps, scorpions, snakes…) are a complex mixture of several agents such as enzymes (phospholipase A2, L-amino acid oxidase, proteases, cysteine-rich secretory proteins), peptides (mastoporan, parabutoporin, disintegrins, etc.) and other toxins [18-20]. Envenomation can cause local and systemic effects characterized by an acute inflammatory reaction with leukocyte recruitment and activation and release of several mediators and cytokines [39-42]. Envenomation is known to be accompanied by egress of neutrophils from the bone marrow into the blood, increasing the number of circulating neutrophils [22, 41]. This phenomenon has also been observed in animal models, as injection of a variety of venoms to mice or rats resulted in an increase of neutrophil population and a massive recruitment to the inoculation site [24, 41, 43]. 

Envenomation is also known to be accompanied by a persistent oxidative stress in bite victims and animal envenomation models [44]. This was evidenced by the presence of lipid peroxidation by measuring the peroxidation product malondialdehyde (MDA) [45-47]. These data suggest a stimulation of ROS production from various sources such as neutrophils. Regarding the effect of envenomation on neutrophil ROS production *in vivo,* data are mainly obtained from the use of animal models. Indeed i.p. injection of *Bothrops asper* (BaV) and *Bothrops jararaca* (BjV) venoms in mice increased phagocytosis and production of hydrogen peroxide (H2O2) in the presence of PMA by polymorphonuclear and mononuclear peritoneal leukocytes [48]. In agreement with this finding, de Souza et al. [49] showed that *Bothrops atrox* snake venom injection in mice induced superoxide production by migrated neutrophils as assessed by nitroblue tetrazolium (NBT) reduction assay. *Bothrops bilineata* snaque venom was able to induce hydrogen peroxide production by human neutrophils [50]. *Echis carinatus* and *Naja naja* snake venoms induced NADPH oxidase activation and NETosis in human neutrophils [51]. It was also shown that *Tityus zulianus* and *Tityus discrepans* scorpion venoms induced hydrogen peroxide production by human neutrophils *in vitro* [52]. Scorpion venom induced ROS production is mediated by TLR4 as administration of a selective inhibitor (TAK-242 or Resatorvid) protected from inflammatory reaction and oxidative stress [53]. Indeed, TLR2, TL4 and CD14 of macrophages were shown to recognize scorpion venom [54].

### Effect of venom constituents on neutrophil ROS production

Effect of venom PLA2

PLA2 cleaves membrane phospholipids at the sn-2-acyl ester bond, releasing arachidonic acid, a powerful inflammatory mediator [55]. Human cells express mainly an 85 kDa cytosolic PLA2 and a 14 kDa secretory PLA2. The cytosolic PLA2 is a key enzyme in neutrophil degranulation and ROS production. Interestingly, PLA2 interacts directly with the phagocyte NADPH oxidase and arachidonic acid itself is able to induce NADPH oxidase activation [56-59]. Several venoms (snake, bee, wasp and scorpion) contain different types of PLA2 [18, 20, 60]. These venom PLA2 are responsible for the inflammatory response induced by the venom [61-64], probably because of the degradation of the plasma membrane and the release of fatty acids such as arachidonic acid. Indeed, venom Asp49 PLA2 from *Bothrops atrox* venom induced degranulation and ROS production in neutrophils but also cytokine production in monocytes and macrophages, and degranulation in mast cells, thus inducing a strong inflammatory reaction [65, 66]. The Lys49-PLA2 from the crude venom of *Crotalus atrox* was reported to induce intracellular calcium increase in human neutrophils [67], a process involved in the stimulation of several functions such as ROS production.

Effect of venom L-amino acid oxidase

L-amino acid oxidase (LAAO) is an enzyme that catalyzes the oxidative deamination of L-amino acids to the corresponding alpha-ketoacids with production of H2O2 and ammonia [68-70]. LAAO is expressed in the venoms of many organisms, including in snakes [71-73]. LAAO has been shown to induce several biological effects such as hemolysis, edema, and activation of inflammatory leukocyte functions [74,75]. In neutrophils, LAAO isolated from snake venom induces chemotaxis, stimulates phagocytosis and release of several mediators [76-78], increasing integrin expression in human neutrophils and activation of other neutrophil functions (ROS production, MPO degranulation, cytokine production and NETs release) [76-78]. Interestingly, Paloschi et al. [79] showed that LAAO from *Calloselasma rhodostoma* snake venom activated NADPH oxidase in neutrophils. 

Effect of venom mastoporan

Mastoparan is a tetradecapeptide toxin found in wasp venoms [80-82]. It was initially characterized as a good inducer of mast cell degranulation [80], and later was found to increase cytosolic calcium concentration and to stimulate IP3 production in human neutrophils [83]. These latter effects could be explained by the direct interaction of mastoparan with Gi proteins and stimulation of the GTPase activity, resulting in PLC activation, IP3 release and cytosolic calcium elevation [84,85]. Mastoporan induces neutrophil chemotaxis, degranulation, CR3 expression and superoxide production [85,86]. In a cell-free system, mastoporan was found to inhibit NADPH oxidase activation by binding to p67phox [87, 88]; however, this inhibitory effect was not observed with intact neutrophils [85, 86]. *In vivo,* mastoporan was able to induce inflammation by increasing TNFα and IL-1β levels and by recruiting neutrophils and macrophages [89].

Effect of venom parabutoporin

Parabutoporin is a peptide produced by *Parabuthus schlechteri*, a South African scorpion species [90]. It was initially known for its antibacterial and antifungal properties [90]. However, it was then shown to also stimulate neutrophil chemotaxis [91,92], degranulation, and to inhibit apoptosis [93,94]. It also inhibits neutrophil superoxide production [91,92], probably through its ability to serve as a PKC substrate, competing with the neutrophil p47phox, thereby inhibiting NADPH oxidase activation [95]. In summary, parabutoporin stimulates some neutrophil functions but inhibits NADPH oxidase activation. Thus, parabutoporin has both pro-inflammatory and anti-inflammatory effects.

Effect of venom disintegrins

Disintegrins are a family of small peptides, most of them containing an RGD (Arg-Gly-Asp) sequence, and are found in snake and other venoms [96,97]. Disintegrins selectively bind to different integrins, such as platelet integrins (alpha IIb, beta 3) to inhibit platelet aggregation, and to neutrophil integrins. Most disintegrins interact with integrins through the RGD sequence loop, resulting in an active site that modulates the integrin activity. It was shown that jarastatin and ocellatusin (two RGD-containing disintegrins) and alternagin-C (a non-RGD-disintegrin), two different disintegrins induced neutrophil migration via integrin activation, but inhibited fMLP- and IL-8-induced neutrophil chemotaxis [98-100]. Jarastatin was also shown to activate ERK1/2 and induce IL-8 expression in neutrophils, while inhibiting apoptosis [99,100]. In contrast to the effects of Jarastatin, Rhodostomin, a different disintegrin, inhibits neutrophil adhesion to fibronectin and ROS production, suggesting an anti-inflammatory effect [101]. VLO5, a disintegrin isolated from Vipera lebetina obtusa venom, was found to activate the A9b1 integrin and to inhibit neutrophil apoptosis by increasing the expression of the proapoptotic protein Bcl2 [102]. Thus, disintegrins have opposite effects on neutrophils, having either a pro-inflammatory or an anti-inflammatory effect.

## Conclusion

Neutrophils are key cells of the innate immunity, modulating the inflammatory reaction. Although they are required for host defense, their excessive activation can lead to excessive release of toxic agents such as ROS that can induce tissue injury and inflammation. Envenomation caused by different animal species (bees, wasps, scorpions, snakes…) is well known to induce a local and acute inflammatory reaction characterized by leukocytes recruitment and activation and the release of several mediators and cytokines. Venom components such as phospholipase A2, L-amino acid oxidase, disintegrins, mastoporan and parabutoporin are able to affect neutrophil ROS production. In this review, we attempted to describe the best characterized effects of the most studied venom components on neutrophil ROS production and the NADPH oxidase activation. Figure 4 summarizes the mechanisms of action of these different molecules on neutrophil pathways. Most venom components have a pro-inflammatory effect, but some can in addition inhibit specific neutrophil functions, exerting both a pro- and anti-inflammatory effects. A multitude of other venom components are known and should be tested on neutrophil functions and pathways and on inflammatory reactions. The venom agents can be used as a powerful tool to modulate neutrophil functions for research or pharmacological purposes.
